# Comparison of health-related quality of life (HRQoL) among healthy, obese and chronically ill Iranian children

**DOI:** 10.1186/s12889-018-6239-2

**Published:** 2018-12-04

**Authors:** Sara Jalali-Farahani, Fahimeh Alsadat Shojaei, Parnian Parvin, Parisa Amiri

**Affiliations:** 1 Research Center for Social Determinants of Health, Research Institute for Endocrine Sciences, Shahid Beheshti University of Medical Sciences, P.O.Box: 19395-4763, Tehran, I. R Iran; 2 Students’ Research Committee, Shahid Beheshti University of Medical Sciences, Tehran, Iran

**Keywords:** Health related quality of life, Obesity, Chronic diseases, Children, Iran

## Abstract

**Background:**

Health-related quality of life (HRQoL) has frequently been compared between both healthy and obese children and healthy and chronically ill children; however, there is glaring lack of evidence regarding comparison of HRQoL in obese children with their counterparts with chronic diseases. Therefore, this study aimed to compare HRQoL among healthy, obese and chronically ill children.

**Methods:**

This cross sectional study was conducted among 802 children **(**8–12 years) who were recruited via convenience sampling method. Participants were 98 healthy, 102 obese and 602 chronically ill children with six groups of chronic conditions including different types of cancer, rheumatoid arthritis, chronic gastrointestinal, kidney, neurologic and respiratory diseases. HRQoL was assessed using the Iranian version of the PedsQL questionnaire and both reports including child self-report and parent proxy-report were obtained. To compare subscales and total scores of HRQoL among healthy, obese and six groups of chronically ill children, the general linear model was used.

**Results:**

Mean self-reported HRQoL total scores were 73.7 ± 13.3 and 74.6 ± 11.8 in girls and boys respectively; based on the parents’ reports, mean HRQoL total scores were 71.6 ± 15.4 and 71.4 ± 13.0 in girls and boys respectively. From the prespectives of both children and parents, HRQoL total score was significantly lower in obese girls compared to both healthy girls and girls with chronic gastrointestinal, kidney, neurologic and respiratory diseases. Considering both children’s and parents’ reports, HRQoL total score was significantly lower in obese boys compared to both healthy boys and boys with chronic respiratory diseases. In terms of subscales of HRQoL, the impairment of HRQoL in obese children, compared to their counterparts with other chronic diseases, was more common in social functioning and physical functioning subscales, specifically in girls.

**Conclusion:**

Obese children reported poorer HRQoL compared to their healthy counterparts, as well as their counterparts with chronic diseases. Current findings emphasize the important impact of childhood obesity on the perceived health of these children, particularly in the social dimension, underscoring thereby the designing, planning and implementation of health promotion programs for prevention and treatment of childhood obesity.

## Background

Obesity has become a serious health problem, as indicated by the significant rise in its prevalence in recent years (1980–2013), among children and adolescents residing in both the developed and the developing countries [1]. In 2015, over a hundred million children were obese worldwide [2]. Obesity has been found to have negative impacts on both the physical and mental health related quality of life of this age group [3]; furthermore, obesity and its related metabolic complications in childhood have the potential to track into adulthood and contribute to the development of type 2 diabetes and cardiovascular diseases [4]. In addition, poorer health-related quality of life (HRQoL) has been reported by obese children and adolescents, compared to their normal weight counterparts [5, 6], a decline has mostly been reported in the physical and social subscales [6].

Health-related quality of life (HRQoL), a multidimensional concept encompassing the physical, psychological and social aspects of health, is the perception of individuals regarding different aspects of their health; it is now considered to be an important health outcome in recent years and can be used for evaluating treatment alternatives, understanding the burden of diseases, identifying health inequalities and allocating resources [7]. There are a number of studies that report impairment of different aspects of HRQoL in children with chronic diseases [8–11]; however, the impaired aspects and the level of impairment were not similar and depended on the type of disease. Considering the aforementioned issues, one beneficial application of HRQoL assessment can be using HRQoL data in identifying the most urgent needs of children with various chronic diseases.

A number of studies have compared HRQoL of obese children with their healthy counterparts and the most of them indicate poorer HRQoL in obese children compared to their healthy counterparts [5, 6]. With the substantial increase in knowledge and awareness related to the grave physical and mental health consequences of obesity in children [3, 4], the old concept of “bigger is better” has changed and nowadays and it is less likely that an ‘obese’ child be considered a ‘healthy’ one. However, the misconception about overweight and obesity is still a common issue among children and their parents [12]. Failing to identify obesity in obese children can potentially influence how their health status is evaluated by themselves and their parents, as demonstrated by the findings of a study conducted in Iran, which reported better HRQoL scores in elementary overweight boys compared to their normal weight counterparts [13]. Moreover, while HRQoL has been frequently compared between healthy and obese children and healthy and chronically ill children, there is limited evidence regarding comparison of HRQoL in obese children with their counterparts with chronic diseases [14]. Furthermore, there is evidence indicating that Iranian obese children and adolescents perceived themselves as healthy individuals and had no concerns about their excessive weight and its complications [15]. Contrary to other chronic diseases, the threat of childhood obesity is usually ignored by families [16]. Therefore, considering the glaring lack of data on this issue, the current study aimed to compare HRQoL in healthy, obese and chronically ill children and provide further insight as to whether impaired HRQoL in obese children is comparable with HRQoL impairment in those who suffer from other chronic diseases.

## Methods

### Participants

The participants of this study were 802 children, aged 8–12 years, who were healthy (*n* = 98), obese (*n* = 102) and those who had been diagnosed with chronic diseases (*n* = 602). Inclusion criteria were age range of 8–12 years and agreement of both the children and their parents to participate in the study. In each study group, if children received any treatment for serious psychological disorders they were excluded. Children with chronic diseases (six disease clusters including cancer, rheumatoid arthritis, chronic gastrointestinal, neurologic, kidney and respiratory diseases) were recruited from teaching hospitals, under coverage of Shahid Beheshti University of Medical Sciences. In addition, healthy and obese children were recruited from medical health centers under coverage of the Shahid Beheshti University of Medical Sciences.

### Data collection

Approval was obtained from the ethics committee of the Research Institute for Endocrine Sciences (RIES) of Shahid Beheshti University of Medical Sciences in Iran. Prior to data collection, both children and parents were informed about the study procedure and its aims and if the child and parent agreed to participate in the study, the parents were asked to sign a written consent form. Assessments in children included anthropometric measurements and completion of the child self-report of the Iranian version of PedsQL™ 4.0 (Pediatric Quality of Life Inventory™ Version 4.0) questionnaire by a trained interviewer [17]; in addition, parents of children were asked to complete self-administered questionnaire which included socio-demographic information and the parent proxy-report of the Iranian version of PedsQL™ 4.0 [17].

### Measurements

Anthropometric measurements included measurement of body weight and height. Body weight was measured using the SECA weighing scale, while the child was without shoes and wore light clothes. Height was measured using SECA body meter while the child was without shoes and stood with shoulders in normal alignment. Using the World Health Organization (WHO) AnthroPlus (version 3.2.2) and macros software, BMI-for-age (BMI Z-score) was determined and obesity was defined as having a BMI Z-score over 2SD [18].

The Iranian version of PedsQL™ 4.0 was used to assess the HRQoL of the children included in the current study; this is a 23-item self-administered questionnaire, which comprises four subscales, i.e. Physical functioning, emotional functioning, social functioning and school functioning [19]. It contains two reports: The children-self report which is completed by the child and the parent-proxy report which is completed by parents. For each item, the respondents chose their answers from among five choices, which range from zero (never a problem) to four (almost always a problem). To score the items, the 0–4 scale was transformed to 0–100 as follows: 0 = 100, 1 = 75, 2 = 50, 3 = 25, 4 = 0, whereby higher scores indicate better HRQoL [19]. The validity and reliability of the Iranian version of PedsQL in Iranian children have been investigated and confirmed previously [17].

### Statistical analysis

Data were statistically analyzed using SPSS software (version 19.0). Descriptive statistics including mean and standard deviation for continues variables and frequency (percentage) for categorical variables were reported. Agreement between adolescent self-reported and parent proxy-reported scores of HRQOL was determined using intra-class correlation coefficient (ICC); an ICC ≤ 0.40 indicates poor to fair agreement, 0.41–0.60 indicates moderate agreement, 0.61–0.80 indicates good agreement and > 0.80 indicates excellent agreement. General linear model (ANCOVA) was used to compare subscales and total scores of HRQoL among healthy, obese and six groups of chronically ill children. To determine significant differences in HRQoL scores between each two groups, pairwise comparison was conducted using the *Bonferroni* Post hoc test. In all models, HRQoL scores were considered as dependent variables and effects of child’s age, parental level of education, maternal working status and father’s job were adjusted. Statistical significance was set at *p* < 0.05.

## Results

Socio-demographic characteristics of children have been presented in Table 1. Mean age of children, their mothers and fathers were 10.2 ± 1.47, 37.7 ± 6.66 and 42.7 ± 7.14 years respectively. A small number of boys (3.3%) and girls (5.3%) were from single parent families. About half the mothers (53.1%) and most fathers (72.2%) had academic degrees; two third of mothers were housewives (67.5%) and the remaining were employed. A limited number of fathers were retired (1.9%), laborers (6.2%) and about one third of them were employees (37.6%). Table 2 compares the socio-demographic characteristics of study participants according to their health status. As indicated in Table 2, mean child’s age and distribution of parental level of education, maternal working status and father’s job differed significantly between children with different health statuses. Hence, in the comparison of HRQoL scores, these characteristics were adjusted in the model.

**Table 1:** Socio-demographic characteristics of the study population by sex groups

	Boys (*n* = 396)	Girls (*n* = 406)	Total (*n* = 802)
Child’s age (y) (*n* = 800)	10.2 ± 1.50	10.2 ± 1.44	10.2 ± 1.47
Mother’s age (y) (*n* = 788)	37.1 ± 6.24	38.4 ± 6.98	37.7 ± 6.66
Father’s age (y) (*n* = 773)	42.0 ± 6.94	43.4 ± 7.27	42.7 ± 7.14
Marital status of parents (*n* = 791)			
-Divorced/widowed	13 (3.3)	21 (5.3)	34 (4.3)
-Married	380 (96.7)	377 (94.7)	757 (95.7)
Level of education of mothers (*n* = 787)			
-Less than high school diploma	63 (16.1)	53 (13.2)	116 (14.7)
-High school diploma	126 (32.7)	127 (31.7)	253 (32.2)
-Academic degrees	197 (51.2)	221 (55.1)	418 (53.1)
Level of education of fathers (*n* = 783)			
-Less than high school diploma	41 (10.6)	33 (8.3)	74 (9.5)
-High school diploma	75 (19.4)	68 (17.2)	143 (18.3)
-Academic degrees	271 (70.0)	295 (74.5)	566 (72.2)
Employment status of mothers (*n* = 788)			
-Housewife	265 (68.5)	267 (66.6)	532 (67.5)
-Employed	122 (31.5)	134 (33.4)	256 (32.5)
Employment status of fathers (*n* = 776)			
-Laborer	26 (6.8)	22 (5.6)	48 (6.2)
-Employee	140 (36.7)	152 (38.5)	292 (37.6)
-Other	207 (54.3)	214 (54.2)	421 (54.3)
-Retired	8 (2.1)	7 (1.8)	15 (1.9)

**Table 2:** Socio-demographic characteristics of the study population by health status

	Healthy (*n* = 98)	Obese (*n* = 102)	Gastro intestinal diseases (*n* = 100)	Kidney diseases (*n* = 100)	Cancer (*n* = 100)	Respiratory diseases (*n* = 101)	Neurologic diseases (*n* = 102)	Rheumatoid arthritis (*n* = 99)	*P* value
Child’s age (y) (*n* = 800)	9.5 ± 1.4	9.5 ± 1.3	10.8 ± 1.6	10.5 ± 1.4	10.2 ± 1.5	10.2 ± 1.4	10.4 ± 1.4	10.6 ± 1.3	< 0.001
Mother’s age (y) (*n* = 788)	37.2 ± 4.7	37.7 ± 5.0	38.7 ± 6.8	37.6 ± 6.0	37.4 ± 7.1	37.9 ± 8.0	37.9 ± 7.9	37.5 ± 7.5	0.839
Father’s age (y) (*n* = 773)	42.1 ± 5.5	41.8 ± 6.2	43.3 ± 6.5	42.2 ± 6.5	42.2 ± 7.2	43.8 ± 8.5	42.9 ± 7.4	43.4 ± 8.7	0.477
Marital status of parents n(%)									
-Divorced/widowed	94(95.9)	96(95.0)	99(100.0)	96(97.0)	95(96.0)	94(94.0)	94(94.0)	89(93.7)	0.399
-Married	4(4.1)	5(5.0)	0(0)	3(3.0)	4(4.0)	6(6.0)	6(6.0)	6(6.3)	
Level of education of mothers n(%)									
-Less than high school diploma	0(0)	3(2.9)	20(20.4)	18(18.6)	11(11.0)	20(20.6)	24(24.2)	19(19.6)	< 0.001
-High school diploma	19(19.8)	30(29.4)	27(27.6)	39(40.2)	35(35.0)	36(37.1)	33(33.3)	34(35.1)	
-Academic degrees	77(80.2)	69(67.6)	51(52.0)	40(41.2)	54(54.0)	41(42.3)	42(42.4)	44(45.4)	
Level of education of fathers n(%)									
-Less than high school diploma	0(0)	0(0)	15(15.2)	16(16.3)	4(4.0)	8(8.3)	17(17.0)	14(15.1)	< 0.001
-High school diploma	3(3.1)	14(14.0)	16(16.2)	21(21.4)	24(24.0)	27(28.1)	19(19.0)	19(20.4)	
-Academic degrees	94(96.9)	86(86.0)	68(68.7)	61(62.2)	72(72.0)	61(63.5)	64(64.0)	60(64.5)	
Employment status of mothers n(%)									
-Housewife	59(61.5)	57(55.9)	72(72.0)	70(71.4)	78(78.0)	70(70.0)	65(65.7)	61(65.6)	0.034
-Employed	37(38.5)	45(44.1)	28(28.0)	28(28.6)	22(22.0)	30(30.0)	34(34.3)	32(34.4)	
Employment status of fathers n(%)									
-Laborer	0(0)	0(0)	9(9.0)	12(12.4)	4(4.0)	3(3.1)	9(9.1)	11(11.8)	0.002
-Employee	34(36.2)	34(35.1)	38(38.0)	41(42.3)	45(45.0)	35(36.5)	36(36.4)	29(31.2)	
-Other	57(60.6)	61(62.9)	53(53.0)	42(43.3)	51(51.0)	57(59.4)	50(50.5)	50(53.8)	
-Retired	3(3.2)	2(2.1)	0(0)	2(2.1)	0(0)	1(1.0)	4(4.0)	3(3.2)	

To determine agreement between adolescent self-report and parent proxy-report of HRQOL scores, ICCs were calculated. The average ICCs for physical functioning, emotional functioning, social functioning, school functioning subscales and total score of HRQOL were 0.81, 0.69, 0.75, 0.70 and 0.85 respectively; these ICC values which ranged between 0.69–0.85, indicate good agreement between the adolescent self-report and the parent proxy-report of HRQOL.

### Self-reported HRQoL scores

Using the general linear model (ANCOVA), adolescent self-reported HRQoL scores were compared among different groups of health status groups after adjusting for child’s age, parental level of education, mother’s working status and father’s job (Tables 3 and 4 for girls and boys respectively). *In girls*, the highest subscales and total scores of HRQoL were reported in healthy girls, whereas the lowest total scores of HRQoL were reported by obese girls; the lowest scores in different subscales of HRQoL, including physical functioning, emotional functioning, social functioning and school functioning were reported by girls with rheumatoid arthritis, cancer, obesity and cancer respectively. *In boys*, except for emotional functioning, the highest subscales and total scores of HRQoL were reported by healthy boys, whereas the lowest total scores of HRQoL were reported by obese boys and boys with rheumatoid arthritis and chronic kidney diseases; the lowest scores in different subscales of HRQoL were reported by boys with rheumatoid arthritis for physical functioning, kidney diseases for emotional and school functioning and obesity for social functioning.

**Table 3:** Self-reported health-related quality of life (HRQoL) subscales and total scores in girls

	Healthy^a^	Obese^b^	Gastro intestinal diseases^c^	Kidney diseases^d^	Cancer^e^	Respiratory diseases^f^	Neurologic diseases^g^	Rheumatoid arthritis^h^	Significant diffrence*	F_7,394_ (*P*value)
Physical functioning	88.0 ± 3.0	59.5 ± 2.8	74.9 ± 2.7	72.8 ± 2.7	63.1 ± 2.8	71.8 ± 2.8	72.5 ± 2.6	58.8 ± 2.8	a-b, a-c, a-d, a-e, a-f, a-g, a-h, c-b, d-b, f-b, g-b, c-e, c-h, d-e, d-h, f-h, g-e, g-h	20.34 (< 0.001)
Emotional functioning	81.5 ± 3.6	66.8 ± 3.3	72.9 ± 3.3	71.3 ± 3.2	57.5 ± 3.4	72.3 ± 3.3	69.7 ± 3.1	63.0 ± 3.4	a-b,a-e, a-g, a-h, c-e, d-e, f-e, g-e	7.92 (< 0.001)
Social functioning	87.1 ± 3.3	59.7 ± 3.1	82.0 ± 3.0	75.9 ± 3.0	68.1 ± 3.2	79.4 ± 3.1	78.5 ± 2.9	72.8 ± 3.2	a-b, a-d, a-e, a-h, c-b, d-b, f-b, g-b, h-b, c-e, e-f	12.48 (< 0.001)
School functioning	88.1 ± 2.7	71.5 ± 2.6	79.3 ± 2.5	75.9 ± 2.5	68.3 ± 2.6	84.4 ± 2.5	74.4 ± 2.4	74.6 ± 2.6	a-b, a-d, a-e, a-g, a-h, f-b, f-e, f-g, f-h, c-e	11.20 (< 0.001)
Total score	86.4 ± 2.3	63.7 ± 2.1	76.9 ± 2.1	73.8 ± 2.1	64.1 ± 2.2	76.3 ± 2.1	73.6 ± 2.0	66.1 ± 2.2	a-b, a-c, a-d, a-e, a-f, a-g, a-h, c-b, d-b, f-b, g-b, c-e, c-h, d-e, d-h, f-e, f-h, g-e, g-h	22.21 (< 0.001)

Note: Analysis of covariance (ANCOVA) test was conducted and child’s age, parental level of education, mother working status and father’s job were adjusted in the model. Adjusted mean and standard error have been reported. a to h: Studied health status. *Results of pairwise comparison

**Table 4:** Self-reported health-related quality of life (HRQoL) subscales and total scores in boys

	Healthy^a^	Obese^b^	Gastro intestinal diseases^c^	Kidney diseases^d^	Cancer^e^	Respiratory diseases^f^	Neurologic diseases^g^	Rheumatoid arthritis^h^	Significant diffrence	F_7,394_ (*P*value)
Physical functioning	90.5 ± 3.0	65.6 ± 2.9	70.3 ± 2.9	69.5 ± 2.7	74.2 ± 2.8	75.3 ± 2.9	77.2 ± 2.7	64.0 ± 2.8	a-b, a-c, a-d, a-e, a-f, a-g, a-h, g-b, f-h, g-h	12.60 (< 0.001)
Emotional functioning	77.0 ± 3.3	71.7 ± 3.3	73.8 ± 3.3	65.2 ± 3.0	68.8 ± 3.2	78.2 ± 3.3	69.2 ± 3.1	68.0 ± 3.1	d-f	2.94 (0.005)
Social functioning	90.5 ± 3.0	70.4 ± 2.9	77.7 ± 2.9	76.0 ± 2.7	82.3 ± 2.8	89.3 ± 2.9	82.0 ± 2.7	78.0 ± 2.8	a-b, a-c, a-d, a-e, a-h, c-f, d-f, f-b, g-b, h-f, b-e	9.16 (< 0.001)
School functioning	85.8 ± 2.6	78.6 ± 2.5	79.3 ± 2.5	72.4 ± 2.3	79.3 ± 2.5	84.7 ± 2.5	76.2 ± 2.4	76.3 ± 2.4	a-d, a-g, d-f	4.57 (< 0.001)
Total score	86.5 ± 2.1	70.8 ± 2.0	74.6 ± 2.0	70.6 ± 1.9	75.9 ± 2.0	81.0 ± 2.0	76.3 ± 1.9	70.6 ± 1.9	a-b, a-c, a-d, a-e, a-g, a-h, b-f, d-f, f-h	12.0 (< 0.001)

Note: Analysis of covariance (ANCOVA) test was conducted and child’s age, parental level of education, mother working status and father’s job were adjusted in the model. Adjusted mean and standard error have been reported. a to h: Studied health status. *Results of pairwise comparison

ANCOVA findings indicated significant differences in the subscales and total scores of HRQoL among girls who were healthy, obese and with other chronic diseases (*p* < 0.001). Similarly, in boys, these findings indicated that mean scores of HRQoL differed significantly among the healthy, obese and those with chronic diseases (*p* < 0.01). To identify significant differences between each two subgroups of study participants, the *Bonferroni* post hoc tests were run, results of which are presented in the last columns of Tables 3 and 4, for girls and boys respectively. As expected, the findings indicate that in both genders, healthy children reported significantly higher HRQoL scores in different subscales, compared to their counterparts with obesity and those with other chronic diseases. In addition, obese girls reported significantly lower HRQoL scores compared to their counterparts with other chronic diseases, including those with gastrointestinal, kidney, respiratory and neurologic diseases in physical functioning, those with rheumatoid arthritis and gastrointestinal, kidney, respiratory and neurologic diseases in social functioning, those with respiratory diseases in school functioning and those with gastrointestinal, kidney, respiratory and neurologic diseases in HRQoL total scores. Furthermore, obese boys reported significantly lower HRQoL scores, compared to their counterparts with other chronic diseases, including those with neurologic diseases in physical functioning, those with cancer, respiratory and neurologic diseases in social functioning and those with respiratory diseases in HRQoL total score. Further details regarding significant differences between each two groups of other chronic diseases are provided in Tables 3 and 4.

### Parent-proxy reported HRQoL scores

Adjusted mean values for parent-proxy reported HRQoL scores in healthy children and those with obesity and various chronic diseases have been estimated using the general linear model (ANCOVA) and are reported in Tables 5 and 6 for girls and boys respectively; based on reported scores, in both genders, the highest subscales and total scores of HRQoL were reported in healthy children, the lowest total scores of HRQoL were reported in girls with cancer and boys with obesity. For subscales of HRQoL in girls, parents reported the lowest scores in girls with obesity for the physical functioning and social functioning subscales and in those with cancer for emotional functioning and school functioning subscales. In terms of subscale scores in boys, parents reported the lowest HRQoL scores in obese boys for the physical and social functioning subscales and in those with kidney diseases for the emotional functioning and school functioning subscales.

**Table 5:** Parent-proxy reported health-related quality of life (HRQoL) subscales and total scores in girls

	Healthy^a^	Obese^b^	Gastro intestinal diseases^c^	Kidney diseases^d^	Cancer^e^	Respiratory diseases^f^	Neurologic diseases^g^	Rheumatoid arthritis^h^	Significant diffrence*	F_7,394_ (*P*value)
Physical functioning	83.5 ± 3.6	53.9 ± 3.4	65.4 ± 3.3	69.9 ± 3.3	54.7 ± 3.5	65.1 ± 3.4	66.7 ± 3.2	56.9 ± 3.5	a-b, a-c, a-d, a-e, a-f, a-g, a-h, b-d, b-g, e-d, e-g, d-h	13.59 (< 0.001)
Emotional functioning	77.0 ± 3.9	59.1 ± 3.6	65.8 ± 3.5	69.5 ± 3.5	44.1 ± 3.7	67.3 ± 3.6	61.6 ± 3.4	63.5 ± 3.7	a-b, a-e, a-g, a-h, b-e, c-e, d-e, f-e, g-e, h-e	11.97 (< 0.001)
Social functioning	84.7 ± 3.2	57.3 ± 3.0	79.6 ± 2.9	78.2 ± 2.9	67.5 ± 3.1	76.0 ± 3.0	77.6 ± 2.8	70.20 ± 3.0	a-b, a-e, a-h, b-c, b-d, b-f, b-g, b-h, c-e, e-d	13.44 (< 0.001)
School functioning	83.4 ± 3.2	68.0 ± 3.0	71.8 ± 2.9	73.7 ± 2.9	61.1 ± 3.0	76.6 ± 2.9	68.3 ± 2.8	69.9 ± 3.0	a-b, a-c, a-e, a-g, a-h, e-c, e-d, e-f	8.47 (< 0.001)
Total score	82.3 ± 2.6	58.9 ± 2.4	70.0 ± 2.4	72.4 ± 2.3	56.6 ± 2.5	70.4 ± 2.4	68.3 ± 2.3	64.0 ± 2.5	a-b, a-c, a-d, a-e, a-f, a-g, a-h, b-c, b-d, b-f, b-g, d-h, e-c, e-d, e-f, e-g	19.05 (< 0.001)

Note: Analysis of covariance (ANCOVA) test was conducted and child’s age, parental level of education, mother working status and father’s job were adjusted in the model

Adjusted mean and standard error have been reported. a to h: Studied health status. *Results of pairwise comparison

**Table 6:** Parent-proxy reported health-related quality of life (HRQoL) subscales and total scores in boys

	Healthy^a^	Obese^b^	Gastro intestinal diseases^c^	Kidney diseases^d^	Cancer^e^	Respiratory diseases^f^	Neurologic diseases^g^	Rheumatoid arthritis^h^	Significant diffrence*	F_7,394_ (*P*value)
Physical functioning	81.8 ± 3.3	56.0 ± 3.2	61.6 ± 3.2	67.4 ± 3.0	66.9 ± 3.2	64.7 ± 3.2	68.4 ± 3.0	59.6 ± 3.1	a-b, a-c, a-d, a-e, a-f, a-g, a-h, b-d, b-g	9.20 (< 0.001)
Emotional functioning	75.9 ± 3.4	64.3 ± 3.3	67.8 ± 3.3	60.6 ± 3.1	68.6 ± 3.2	72.5 ± 3.3	64.4 ± 3.1	68.1 ± 3.2	a-b, a-d, d-f	3.38 (0.002)
Social functioning	84.6 ± 3.3	63.5 ± 3.3	73.8 ± 3.3	68.6 ± 3.0	74.8 ± 3.2	79.3 ± 3.2	75.7 ± 3.0	69.3 ± 3.1	a-b, a-d, a-h, b-e, b-f, b-g	6.91 (< 0.001)
School functioning	81.7 ± 2.8	75.0 ± 2.7	70.0 ± 2.7	63.3 ± 2.5	71.4 ± 2.6	77.2 ± 2.7	68.5 ± 2.5	66.5 ± 2.6	a-c, a-d, a-e, a-g, a-h, b-d, d-f, f-h	6.73 (< 0.001)
Total score	81.1 ± 2.3	63.6 ± 2.3	67.4 ± 2.4	65.3 ± 2.1	70.0 ± 2.2	72.2 ± 2.3	69.1 ± 2.1	65.1 ± 2.2	a-b, a-c, a-d, a-e, a-f, a-g, a-h, b-f	9.21 (< 0.001)

Note: Analysis of covariance (ANCOVA) test was conducted and child’s age, parental level of education, mother working status and father’s job were adjusted in the model

Adjusted mean and standard error have been reported. a to h: Studied health status. *Results of pairwise comparison

Based on parents’ reports, findings of ANCOVA indicated that in both genders, HRQoL subscale and total scores differed significantly among children who were healthy, obese and those with other chronic diseases (*p* < 0.01). To identify significant differences between each two subgroups of participants, *Bonferroni* post hoc tests were run and the results are presented in the last columns of Tables 5 and 6 for girls and boys respectively; as expected, these findings indicate that parents reported significantly higher HRQoL scores in different subscales for healthy children, compared to their counterparts with obesity and other chronic diseases. In addition, parents reported significantly lower HRQoL scores for obese girls compared to their counterparts with other chronic diseases, including those with kidney and neurologic diseases in physical functioning, those with rheumatoid arthritis, gastrointestinal, kidney, respiratory and neurologic diseases in social functioning and those with gastrointestinal, kidney, respiratory and neurologic diseases in HRQoL total scores. Furthermore, parents reported significantly lower HRQoL scores for obese boys, compared to their counterparts with other chronic diseases, including those with kidney and neurologic diseases in physical functioning, those with cancer, neurologic and respiratory diseases in social functioning and those with respiratory diseases in HRQoL total scores.

## Discussion

The current study aimed to compare HRQoL scores in healthy, obese and chronically ill Iranian children. Findings indicate that healthy chidlren had the highest HRQoL scores, while obese children and those with different chronic diseases had the lowest scores in different subscales of HRQoL. In addition, there were significant differences in HRQoL among participants with different types of chronic diseases; moreover, obese children reported poorer HRQoL, compared to both their healthy counterparts and those with other chronic diseases.

### HRQoL in children with chronic diseases compared to healthy ones

Findings of the current study indicate that healthy children had the highest HRQoL scores among study groups and children with chronic diseases had significantly poorer HRQoL, compared to their healthy counterparts, particularly in their physical, social and school functioning subscales. Few studies have investigated general functioning of chronically ill children and the current findings indicate significant decline in the general HRQoL in children with various chronic diseases across different subscales. These findings are consistent with those of previous studies which report better HRQoL in healthy children, compared to those with other chronic diseases, including epilepsy [8], migraine [20], rheumatic diseases [9], mild to moderate chronic kidney disease and end-stage renal disease (ESRD) [10, 21], irritable bowel syndrome (IBS) and inflammatory bowel disease (IBD) [11, 22].

### HRQoL in obese children compared to their healthy counterparts

Findings of the current study indicate that, except for the self-reported emotional functioning and school functioning subscale in boys, both obese boys and girls had poorer HRQoL scores compared to their healthy counterparts; these findings are in line with those of previous studies which report poorer HRQoL in both clinical [23, 24] and community samples of obese children and adolescents [25, 26]. Similarly, most previous studies from Iran, report lower HRQoL scores in overweight and obese children and adolescents, compared to their normal weight counterparts, specifically in older age groups, including adolescents [25, 27, 28]. According to data available, the decline in HRQoL has been demonstrated in different subscales [23, 26] and, based on a review, it was frequently reported, specifically in the physical and social functioning subscales [6], results in agreement with the findings of the current study.

### HRQoL in children with chronic diseases compared to their obese counterparts

An interesting finding of the current study was the reporting of poorer HRQoL by obese children, compared to their counterparts with chronic illnesses, a finding however comparable to the limited data available [14, 29]. Further deterioration in the HRQoL of obese children, compared to their counterparts with chronic diseases in the current study, was observed specifically in their physical and social functioning. Poorer physical health in obese children, compared to their counterparts with other chronic diseases may be due to the clustering of various risk factors in obese children; in other words, previous findings indicate that childhood obesity is accompanied by several health problems such as cardivascular disease risk factors, breathing problems including asthma and sleep apnea, joint problems and musculoskeletal discomfort, as well as fatty liver disease, gallstones, and gastro-esophageal reflux [30–33] and that obese children may suffer from several immediate health risks simultaneously, whereas in other chronic conditions such as asthma, the ill child may encounter only one problem at a time. Moreover, in terms of poorer social functioning in obese children, this impairment can be explained mainly by peer victimization, teasing, weight stigmatization, and less acceptance by their peer group, which can result in self esteem problems, social isolation and marginalization and could negatively influence social interaction of these children [34–36]. The current findings prioritize obese children as a group for interventions, aiming at improving their HRQoL in children. Furthermore, additional interventions targetting the physical and social wellness of obese children may also be necessary.

Current findings also indicate that obese boys appear to fare better in their HRQoL assessments compared to those of their healthy and chronically ill counterparts than obese girls did compared to their healthy and chronically ill counterparts; these gender differences may be due to different beliefs regarding excessive weight in girls and boys. While smaller and slimmer bodies are preffered by girls, larger and muscular bodies are preferred by boys; this different ideal body size and shape in boys and girls may be one of the reseaons for observed gender differences [37]. Additionally, based on the findings of a previous study conducted among Tehranian adolescents, some overweight/obese boys believed that they are more resistant to illness and physical blows and had positive self-images compared to their normal weight peers; they also believed that they have equal ability to compete in sports and physical activities with their normal weight peers [15]. The abovementioned beliefs may be responsible for the reporting of better HRQoL scores in obese boys compared to obese girls.

### Strengths and limitations

Among its strengths, this study is among the first attempts providing information regarding the HRQoL of Iranian children across different chronic health conditions and comparing the HRQoL of obese children with their counterparts with different chronic diseases. In addition, using a generic quality of life instrument allowed us to compare HRQoL among different groups including healthy, obese and chronically ill children. Besides, using PedsQL as one of the most common instruments for assessment of HRQoL in different countries, facilitated the comparision of our findings across various populations with different cultures; moreover, as this scale includes both the child’s self-report and the parent proxy-report, it facilitates the chance of obtaining the reports of both the children and their parents. Of course, our study has its limitations; low prevalence rates of most chronic pediatric diseases and the sample size did not allow us to match samples for age and socio-economic factors. Furthermore, not considering the duration and severity of diseases was another limitation of the current study which should be considered in future research on the topic.

## Conclusion

In summary, these findings highlight certain pediatric populations which may need to be prioritized for interventions targetting their HRQoL. To conclude, obese children reported lower HRQoL scores, compared to healthy children. Moreover, HRQoL scores reprted by obese children were similar to girls with cancer and boys with rheumatoid arthritis; additionally, in different subscales of HRQoL, obese children reported poorer HRQoL, compared to those with chronic diseases, including gastrointestinal, kidney, respiratory and neurologic diseases, cancer and rheumatoid arthritis. These findings emphasize the impact of childhood obesity on their self perceived health and also implies the necessity of designing, planning and implementing health promotion strategies/programs for the prevention and treatment of childhood obesity.

## Abbreviations

BMI: Body mass index
HRQoL: Health-related quality of life
PedsQL™ 4.0: Pediatric Quality of Life Inventory™ Version 4.0
